# Investigating the culturable atmospheric fungal and bacterial microbiome in West Texas: implication of dust storms and origins of the air parcels

**DOI:** 10.1093/femsmc/xtaa009

**Published:** 2020-12-15

**Authors:** Moamen M Elmassry, Nandini Ray, Sara Sorge, Jennifer Webster, Kyle Merry, Angelica Caserio, Daniel J Vecellio, Cassandra Kruczek, Scot Dowd, Karin Ardon-Dryer, Jennifer Vanos, Michael J San Francisco

**Affiliations:** Department of Biological Sciences, Texas Tech University, Lubbock, TX 79409, USA; Department of Biological Sciences, Texas Tech University, Lubbock, TX 79409, USA; Department of Biological Sciences, Texas Tech University, Lubbock, TX 79409, USA; Department of Biological Sciences, Texas Tech University, Lubbock, TX 79409, USA; Department of Biological Sciences, Texas Tech University, Lubbock, TX 79409, USA; Department of Biological Sciences, Texas Tech University, Lubbock, TX 79409, USA; Department of Geography, Texas A&M University, College Station, TX 77843, USA; Department of Medical Education, Texas Tech University Health Sciences Center, Lubbock, TX 79430, USA; Molecular Research LP, Clovis Road, Shallowater, TX 79363, USA; Department of Geosciences, Atmospheric Science Group, Texas Tech University, Lubbock, TX 79409, USA; School of Sustainability, Arizona State University, Tempe, AZ 85281, USA; Department of Biological Sciences, Texas Tech University, Lubbock, TX 79409, USA; Honors College, Texas Tech University, Lubbock, TX 79410, USA

**Keywords:** fungi, bacteria, atmospheric microbiome, air parcels, dust storms, West Texas, allergens

## Abstract

Individuals often experience ailments such as allergies, asthma and respiratory tract infections throughout the year. Weather reports often include estimations of common allergens that can affect these individuals. To describe the local ‘atmospheric microbiome’ in Lubbock, Texas, USA, we examined the culturable fungal and bacterial microbiome present in the air on calm and dust storm days using internal transcribed spacer (ITS)-1 and 16S rRNA amplicon sequencing, respectively. While some types of airborne fungi were frequently present throughout the year, distinct differences were also observed between calm and dust storm days. We also observed the influence of the origin of air parcels and wind elevation of the air trajectory. The most abundant genera of fungi identified during the study period were *Cryptococcus, Aureobasidium, Alternaria, Cladosporium* and *Filobasidium*. This observation was not surprising considering the agricultural intensive environment of West Texas. Interestingly, *Cladosporium*, a common allergenic mold, was increased during days with dust storm events. The predominant bacterial genera observed were *Bacillus, Pseudomonas, Psychrobacter, Massilia* and *Exiguobacterium*. The relative abundance of the psychrophiles, *Psychrobacter* and *Exiguobacterium*, was surprising, given the semi-aridity of West Texas. Coupling our observations with back trajectories of the wind (Hybrid Single-Particle Lagrangian Integrated Trajectory models) demonstrated that dust storms, regional anthropogenic activity and origin of air parcels are important influences on the diversity and temporal presence of the atmospheric microbiome.

## Teaser

Dust storms impact the atmospheric microbiome; for example they increase the abundance of Cladosporium, an allergenic fungus that may affect the health of susceptible individuals.

## INTRODUCTION

The total mass of the atmosphere is ∼5.14 × 10^18^ kg (Trenberth and Guillemot 1994). The atmosphere is mainly constituted of gases and particulate matter (PM) of different sizes. A constant part of our life as humans is the direct exposure to air. Although airborne microorganisms represent a significant part of our surrounding fungal and bacterial microbiome, they are greatly underestimated and understudied (Meadow *et al*. 2015; Fujiyoshi, Tanaka and Maruyama 2017). This continuous exposure may have beneficial and detrimental effects, which need to be investigated carefully (Prussin and Marr 2015).

Recent work has focused on characterizing the upper troposphere microbiome and its potential role in cloud formation (DeLeon-Rodriguez *et al*. 2013). Other studies have attempted to investigate the built environment ‘indoor-air’ microbiome, including homes (Dunn *et al*. 2013), university buildings (Meadow *et al*. 2014), urban subway systems (Leung *et al*. 2014) and hospitals (Tong *et al*. 2017). The effect of dust storms on the atmospheric microbiome was examined by Mazar *et al*. (2016). It was found that dust storms influence the airborne microbiome to become dominated by the soil-associated microbiome instead of the anthropogenic-associated microbiome (Mazar *et al*. 2016; Marone *et al*. 2020). Several reports have suggested that thunderstorms are linked to an increase in respiratory problems (e.g. asthma), which were linked to allergens (i.e. fungal spores or airborne pollens) transported by thunderstorms (Nasser and Pulimood 2009; Yair *et al*. 2019).

Discussions have been raised regarding the potential role of the atmospheric microbiome on global health (Hanson *et al*. 2016; Gat *et al*. 2017). The average human breathes in ∼20 000 L of air every day. For individuals with chronic respiratory disorders, including asthma, cystic fibrosis and other lung infections, microbes and allergens suspended as PM in the air can negatively impact their health (Douwes *et al*. 2003). Particulate matter is a mixture of solid particles of different sizes (2.5, 10 and >10 μm designated PM_2.5_, PM_10_, respectively), and minute liquid droplets known as aerosols, some of which like dust and smoke are visible to the naked eye when present at high concentrations. Bioaerosols, however, are microscopic fragments suspended in the air and are present in particles like pollen, and microbes (bacteria, fungi and viruses), which may be pathogenic and/or allergenic (Fischer and Dott 2003; Haas *et al*. 2013). Suspended PM can remain in the atmosphere for extended periods of time, with smaller and lighter particles (PM_2.5_) transported by the wind for extended time periods and over a long distances (thousands of kilometers) (Kim, Kabir and Kabir 2015). Thus, it is important to leverage biometeorological prediction models to identify the origin of air parcels as well as the wind direction and particle concentration. Information on the atmospheric microbiome is also important due to their potential impact on cloud formation (Spracklen and Heald 2014) and human health (Griffin 2007).

The City of Lubbock, Texas is located in a semi-arid region, and thus has conditions conducive to dust storms due to wind erosion of dry surface soils. Our study site at Texas Tech University is surrounded by millions of acres of cattle, dairy farms, cotton fields and vineyards. Dust storms are responsible for the majority of PM in the local environment (Neff *et al*. 2013; Kelley *et al*. 2020). High levels of PM influence bioaerosol generation and dispersal, and increased concentrations of PM and bioaerosols generally track together (Adhikari *et al*. 2006). Bacteria tend to be associated with coarser particles, while fungal spores are associated with smaller particles (Haas *et al*. 2013). Inhalation of bioaerosols can lead to lung irritation, respiratory illnesses and susceptibility to viral, fungal and bacterial pathogens (Douwes *et al*. 2003). Several airborne fungi such as *Cladosporium, Penicillium, Aspergillus* and *Alternaria* have been implicated in allergies (Levetin *et al*. 2016). For example, *Alternaria alternata* can exacerbate asthma and allergy flare-ups (Denning *et al*. 2006; Levetin *et al*. 2016). Moreover, the abundance of airborne *Cladosporium* and *Alternaria* was correlated with asthma exacerbation episodes (Fukutomi and Taniguchi 2015). Numerous health studies in the last two decades have linked high concentrations of particulate air pollution with an increase in emergency room visits, hospital admissions and even premature death (Anderson, Thundiyil and Stolbach 2012; Karanasiou *et al*. 2012). Epidemiological studies have linked PM prevalence to the number of daily deaths and hospitalizations, most likely as a result of respiratory and cardiovascular diseases (Karanasiou *et al*. 2012). According to Cohen *et al*. (2004), PM_2.5_ (particulate matter with aerodynamic diameter ≤2.5 µm) are responsible for ∼1.4% of deaths worldwide (Cohen *et al*. 2004). In order for healthcare workers to adequately address their patients’ needs, they need to know which types of microbes may be present as a result of changes in weather patterns. The physicians at the Texas Tech University Health Sciences Center note that the majority of their patients with asthma are seen following high-wind episodes, and information on airborne microbes could provide valuable information when treating these patients (Tarbox, pers. comm. 2016).

In order to describe the local ‘atmospheric microbiome’ in Lubbock, TX, a total of 47 air samples were collected on 22 days from September 2015 through November 2016. Meteorological information during the sampled days was collected from the local airport meteorological station, while the origins of the air parcels were tracked using Hybrid Single-Particle Lagrangian Integrated Trajectory (HYSPLIT) back trajectory analyses. To examine the atmospheric microbiome, we used a modified approach by culturing the bacteria and fungi prior to amplicon [internal transcribed spacer (ITS)-1] or 16S rRNA gene amplicon next-generation sequencing to identify fungal and bacterial genera that comprised these communities. We also examined the influence of dust storm events on the diversity and composition of the atmospheric microbiome.

## MATERIALS AND METHODS

### Sample collection

Forty-seven samples across 22 days were collected at Texas Tech University campus (33° 35' 5.4456'' N, 101° 52' 41.178'' W) at ground level and ∼60 m (Figure S1, Supporting Information)] over a period from September 2015 to November 2016 in Lubbock, TX (Table S1, Supporting Information). All samples were obtained between 4:00 and 6:00 p.m. local standard time (LST), which is when soil moisture and relative humidity are known to be the lowest in the air, and there is sufficient time for the air to mix with the high concentrations of microbes and dust that can impact humans (Tong 1999). Samples were collected at least once a month during calm days (control), and experimental samples were taken when dust storms (wind speeds > 48km/h) occurred.

### Fungal and bacterial atmospheric microbiome sample collection

Previous studies investigating the atmospheric microbiome have used culturing techniques or direct next-generation sequencing of air samples to analyze the atmospheric microbiome. Limitations of culturing techniques or direct next-generation sequencing are well-known (Stephens *et al*. 2015). We chose to investigate the culturable microorganisms only in order to eliminate the possibility of sequencing DNA artifacts carried by the air. Sampling was conducted using Petri plates containing potato dextrose agar (PDA) (Thermo-Fisher Scientific, Waltham, MA, USA) or tryptic soy agar (TSA) (Thermo-Fisher Scientific, Waltham, MA, USA). The plates were held open into the incoming air for 2 min to collect airborne fungi and bacteria. The plates were then incubated at room temperature for 48 h and stored at 4°C until sequencing. Originally, 47 samples for 22 days were collected using both PDA and TSA media. The number of replicates per day varied from 2 to 4. However, some samples were discarded because of their low-read quality after sequencing and that they would not represent the true microbial diversity in the environment. Therefore, we analyzed the culturable fungal microbiome using 47 replicates collected from 22 days, while for the culturable bacterial microbiome, 36 replicates collected from 17 days were used.

### ITS and 16S rRNA DNA sequencing and analysis

DNA extraction and amplicon-specific sequencing were performed using the previously collected plates by Molecular Research LP (MR DNA), Shallowater, TX, USA as described at MR DNA Laboratory (www.mrdnalab.com). The ITS-1 rRNA region was targeted to characterize the fungal community, while the 16S rRNA gene V4 variable region was used to identify the bacterial community. Sequencing was performed on Illumina MiSeq platform following the manufacturer's guidelines. Raw sequencing data were deposited under BioProject accession number PRJNA637810 in the National Center for Biotechnology Information BioProject database. ITS-1 and 16S rRNA sequence data were analyzed using QIIME 2 (Bolyen *et al*. 2019). In summary, sequences were filtered, denoised and joined. Then, DADA2 plugin was used within QIIME 2 to determine exact amplicon sequence variants (ASVs). For taxonomy assignment of ITS and 16S rRNA ASVs, SILVA 132 (Pruesse, Peplies and Glöckner 2012) and UNITE 7.2 (Community 2017) databases were used, respectively.

### Back trajectory analysis and meteorological information

In order to determine the link between the microbial species present within the local environment and the origin of the air mass moving through the area, back trajectories and surface meteorological variables were used to determine the atmospheric conditions that result in high levels of specific fungi and bacteria. The HYSPLIT model (https://www.arl.noaa.gov/) was used to create backward trajectories of air parcels moving through the air over 72-hour periods from the coordinates of the collection site. Using a Lagrangian framework to time-integrate air parcel advection backward through the domain, HYSPLIT outputs 3D paths of atmospheric transport based on observational and model data (Draxler and Hess 1997). The Eta Data Assimilation System 40 km (EDAS40) dataset was used to force the HYSPLIT runs. Back trajectories at 500 m above ground level were used to capture the conditions that could be expected to be representative of a well-mixed daytime boundary layer.

Meteorological information, such as hourly ambient temperature, relative humidity, wind speed, wind direction, wind gust, visibility, station pressure and precipitation, was retrieved from the local National Weather Service (NWS 2019) meteorological station, which is located at the Lubbock Preston Smith International Airport (33° 39' 48.96'' N, 101° 49' 22.8'' W), ∼9.8 km north from the sample collection. Dust storm days were identified using the station Present Weather Code. Information of main meteorological conditions of each sample can be found in Table S1 (Supporting Information).

## RESULTS

### The culturable atmospheric fungal microbiome

**Figure 1.**
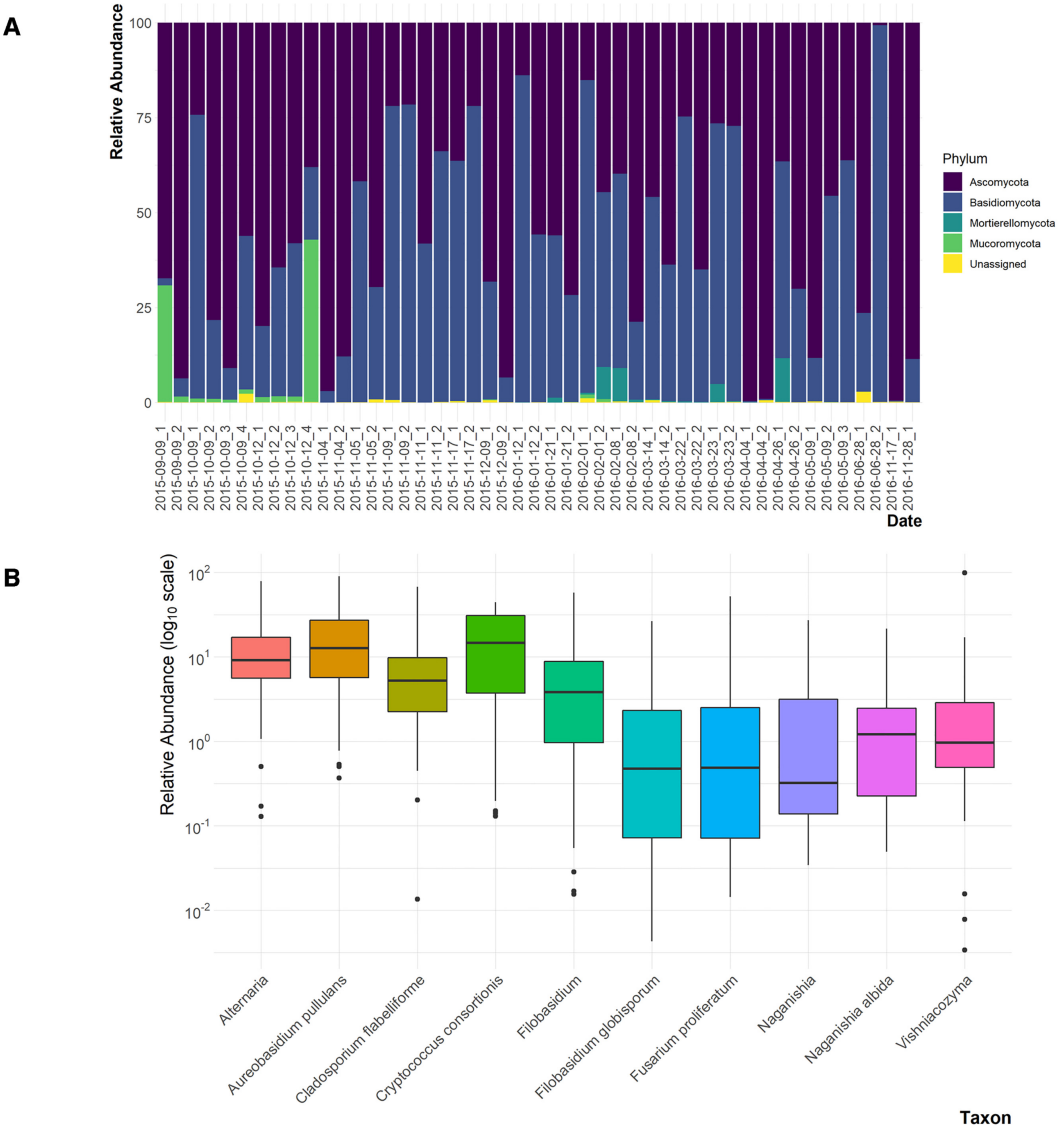
The culturable atmospheric fungal microbiome. **(A)** A stacked bar plot shows phyla belong to fungi. **(B)**Most abundant genera and species of the culturable atmospheric fungal microbiome. Relative abundance of the top taxa with an average relative abundance ≥ 1% shown as boxplot.

Across the analyzed fungal microbiome samples, 695 ASVs were identified, with a mean frequency of ∼ 112,753 (range: 29 676–326 859). Ascomycota and Basidiomycota were the top two prevalent phyla among all samples with an average relative abundance 57% and 40%, respectively (Fig. 1A). Other phyla (e.g. Mucoromycota and Mortierellomycota) were found with an average relative abundance below 5% (Fig. 1A). Among the most resolved ASVs (at the species and genus levels), four genera and six species had the most abundant average relative abundance ≥1% (Fig. 1B). The most abundant taxa were *Aureobasidium pullulans* (with an average relative abundance of ∼18%), *Cryptococcus consortionis* (∼17%), *Alternaria* (∼16%), *Cladosporium flabelliforme* (10%) and *Filobasidium* (∼8%) (Fig. 1B).

### The culturable atmospheric bacterial microbiome

**Figure 2.**
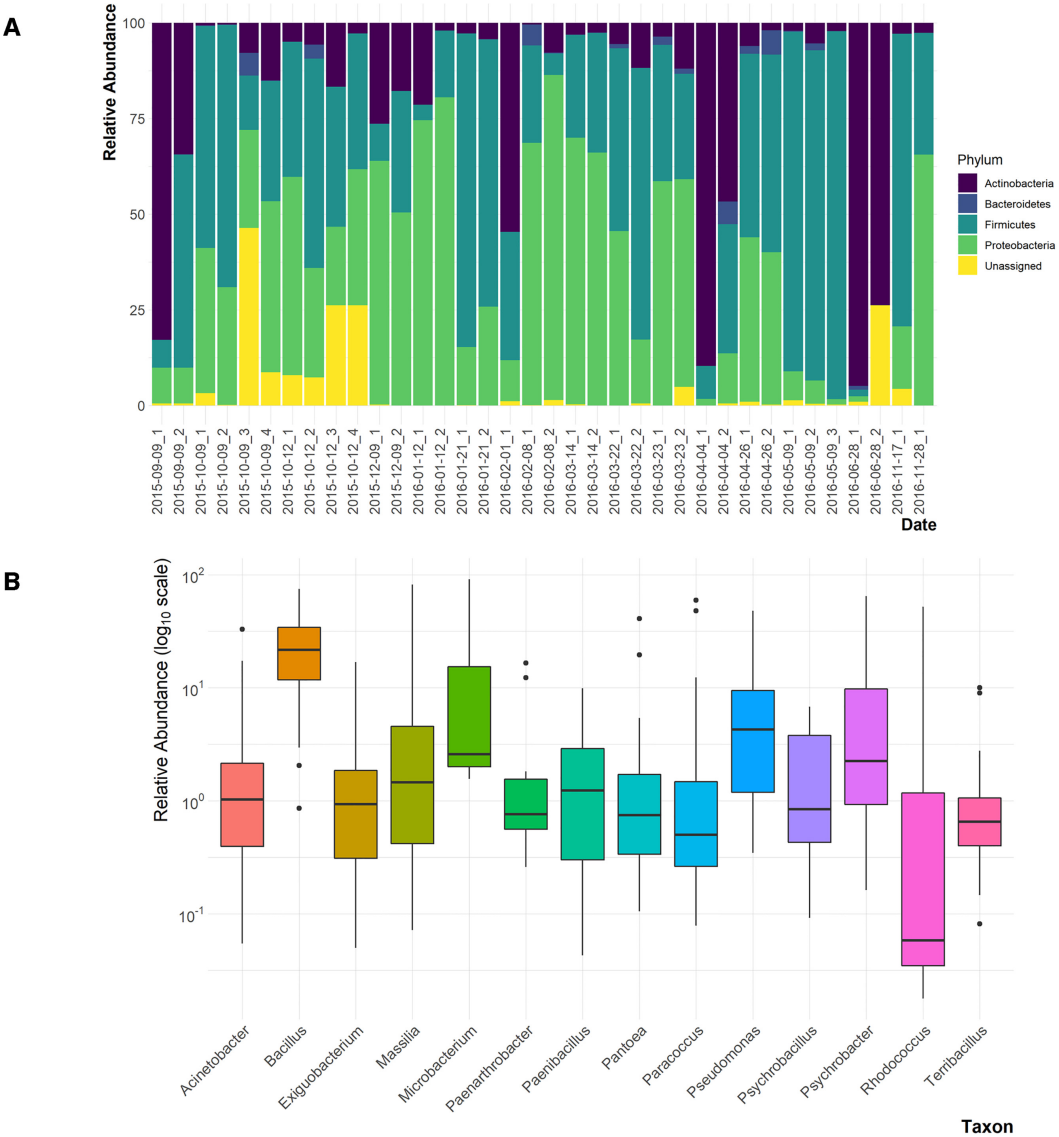
The culturable atmospheric bacterial microbiome. **(A)** A stacked bar plot shows phyla belong to bacteria. **(B)** Most abundant genera and species of the culturable atmospheric bacterial microbiome. Relative abundance of the top taxa with an average relative abundance ≥ 1% shown as boxplot.

Across the analyzed bacterial microbiome samples, 1,153 ASVs were identified, with a mean frequency of ∼39,158 (range: 10 012–22 754). The most abundant bacterial phyla were Firmicutes, Proteobacteria, Actinobacteria and Bacteroidetes with average relative abundance of 40%, 35%, 19% and 1%, respectively (Fig. 2A). Among the most resolved ASVs (at the genus level), 10 genera had the highest average relative abundance ≥1% (Fig. 2B). The most abundant taxa were *Bacillus* (with an average relative abundance of ∼26%), *Pseudomonas* (∼9%), *Psychrobacter* (∼8%) and *Massilia* (∼5%) (Fig. 2B).

### The impact of dust storm events and the origin of the air on the local atmospheric microbiome

**Figure 3.**
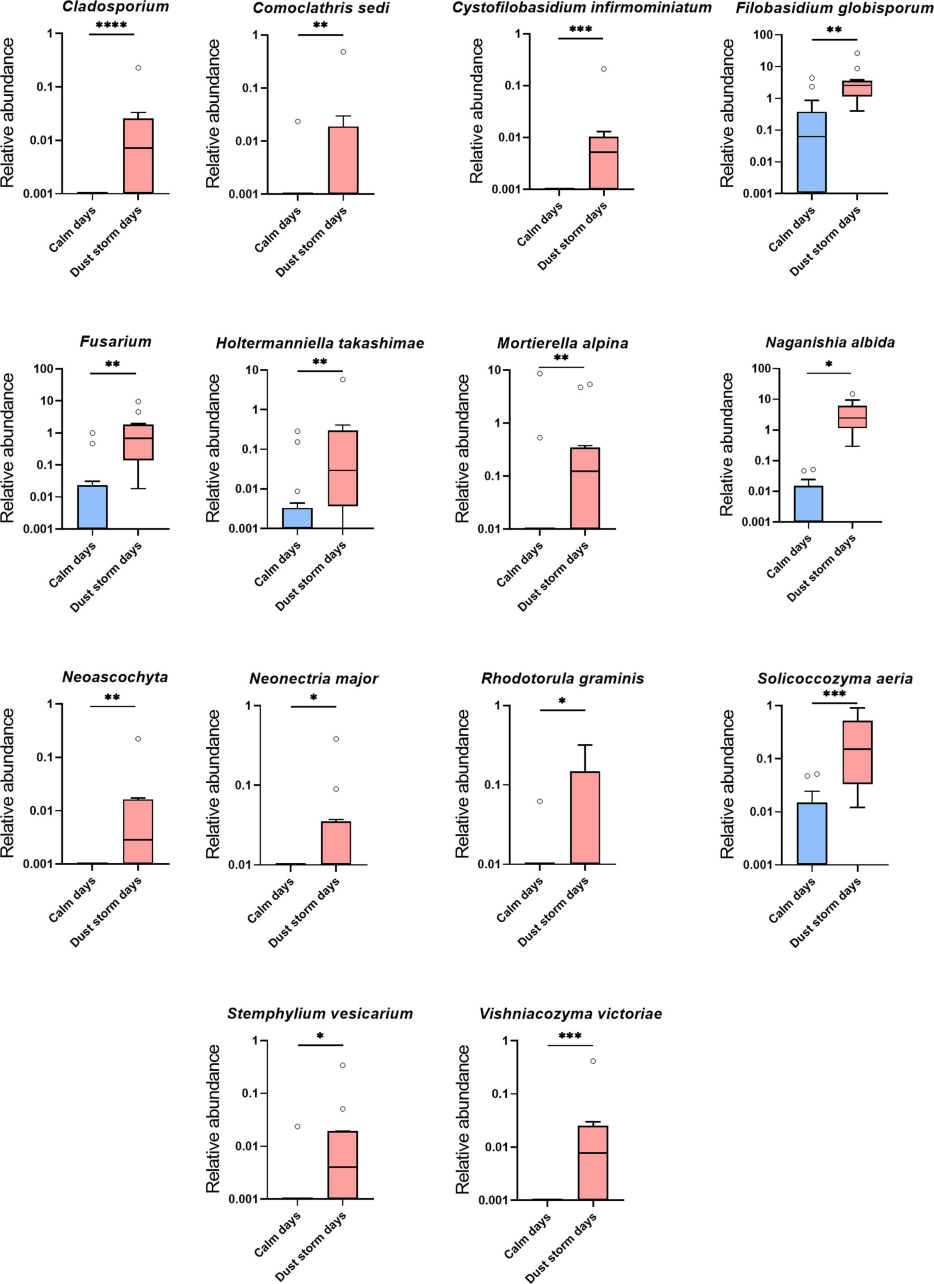
The impact of dust storms on the relative abundance of the culturable atmospheric fungal microbiome. Genera and species of fungal microbiome with statistical significance in abundance between calm and dust storm days are shown (*P < 0.05, **P < 0.01, ***P < 0.001, ^****^P < 0.0001). The Wilcoxon Rank-Sum test was used to determine statistical significance, followed by the Benjamini–Hochberg procedure to correct the *P* values. Data are represented as boxplots, which extend from the 25th to 75th percentiles and the line in the middle of the box represents the median.

**Figure 4.**
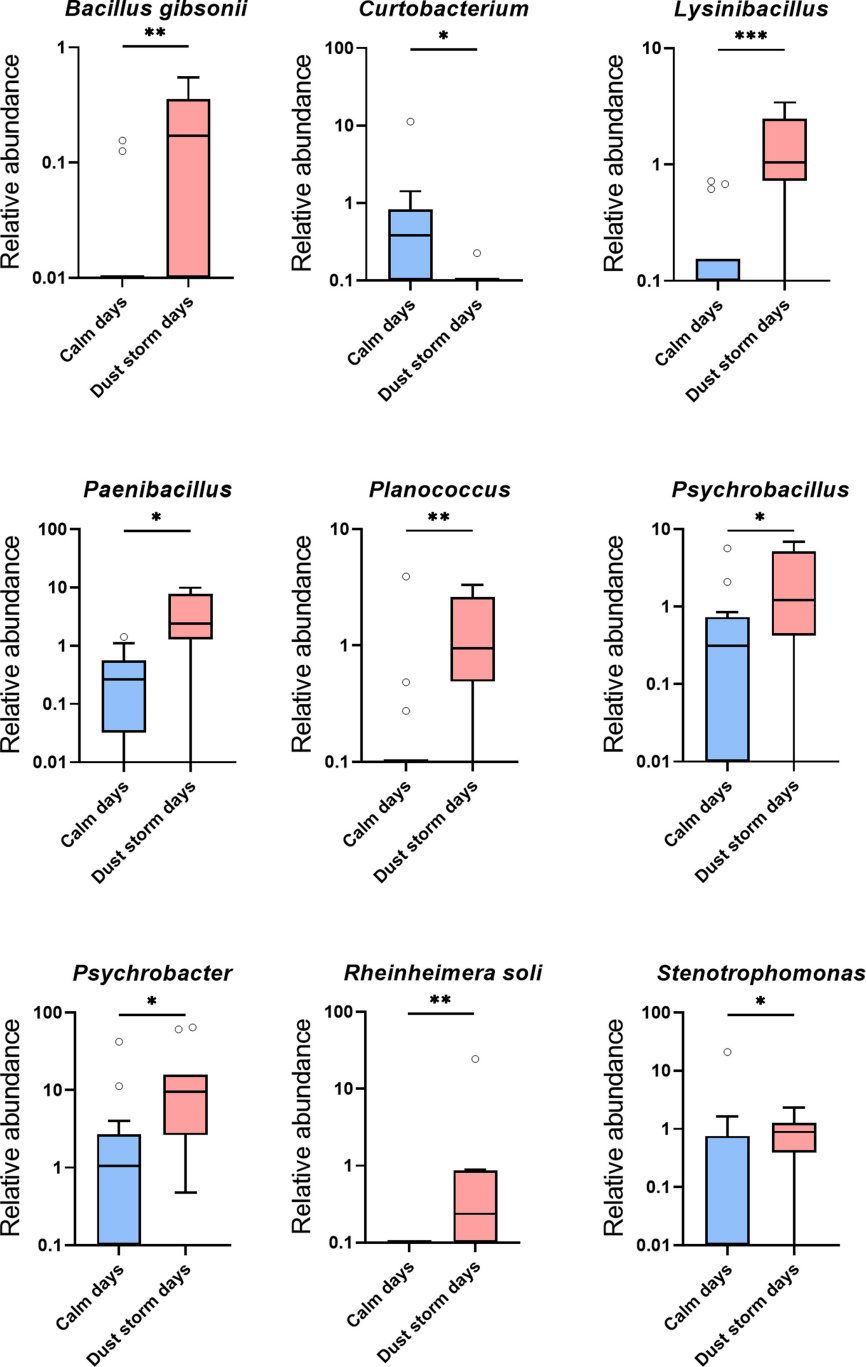
The impact of dust storms on the relative abundance of the culturable atmospheric bacterial microbiome. Genera and species of bacterial microbiome with statistical significance in abundance between calm and dust storm daysare shown (*P < 0.05, **P < 0.01, ***P < 0.001, ^****^P < 0.0001). The Wilcoxon Rank-Sum test was used to determine statistical significance, followed by the Benjamini–Hochberg procedure to correct the *P* values. Data are represented as boxplots, which extend from the 25th to 75th percentiles and the line in the middle of the box represents the median.

**Figure 5.**
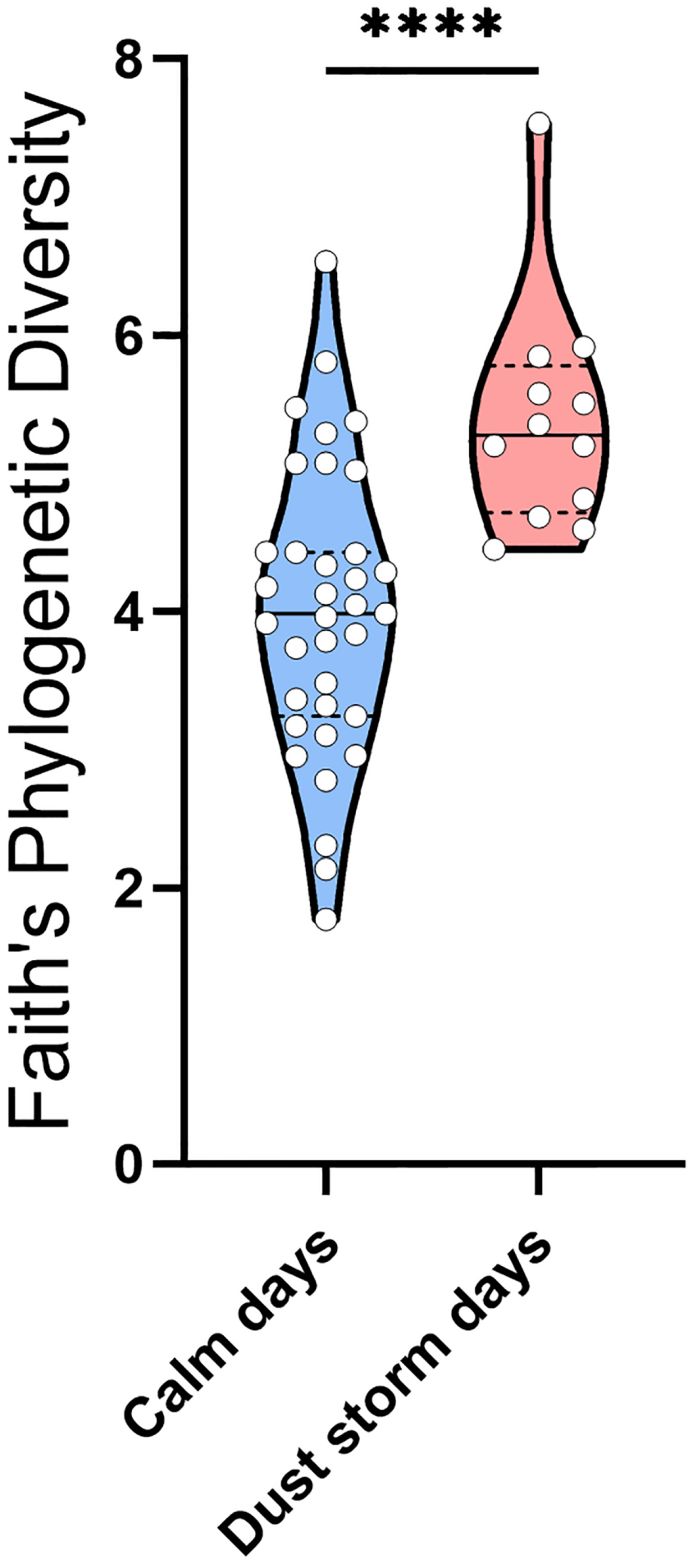
The impact of dust storms on the alpha diversity (richness) of the culturable atmospheric fungal microbiome. The non-parametric Mann–Whitney *U* test was used to determine statistical significance. Genera and species of fungal microbiome with statistical significance in abundance between calm and dust storm days (^****^P < 0.0001). Data are represented as violin plot. The line in the middle of the box represents the median

One of the goals of the current study was to examine the effect of dust storm events on the atmospheric microbiome. To do so, we used a subset of the samples collected during days of blowing dust storms, identified by the NWS station, in comparison to calm days. Six high-wind days with dust storms with a median wind speed of ∼48.3 km/h and seven calm (control) days with a median speed of 22.5 km/h were identified. We detected 14 fungal genera and species (Fig. 3) and 9 bacterial genera and species (Fig. 4) whose relative abundance was significantly increased by dust storms (except for *Curtobacterium* that was decreased). This analysis was performed using Wilcoxon rank-sum test with Benjamini–Hochberg false discovery rate correction. Fungal taxa included *Neonectria major, Rhodotorula graminis, Cystofilobasidium infirmominiatum, Filobasidium globisporum, Naganishia albida, Solicoccozyma aeria, Holtermanniella takashimae, Vishniacozyma victoriae, Mortierella alpine, Fusarium, Comoclathris sedi, Stemphylium vesicarium, Cladosporium* and *Neoascochyta* (Fig. 3). Bacterial taxa included *Curtobacterium, Bacillus gibsonii, Paenibacillus, Lysinibacillus, Planococcus, Psychrobacillus, Rheinheimera soli, Psychrobacter* and *Stenotrophomonas* (Fig. 4). We also observed that the richness of the fungal microbiome using Faith's phylogenetic diversity was significantly increased in dust storm days (Fig. 5), but no change was observed in the richness of the bacterial microbiome between calm and dust storm days.

**Figure 6.**
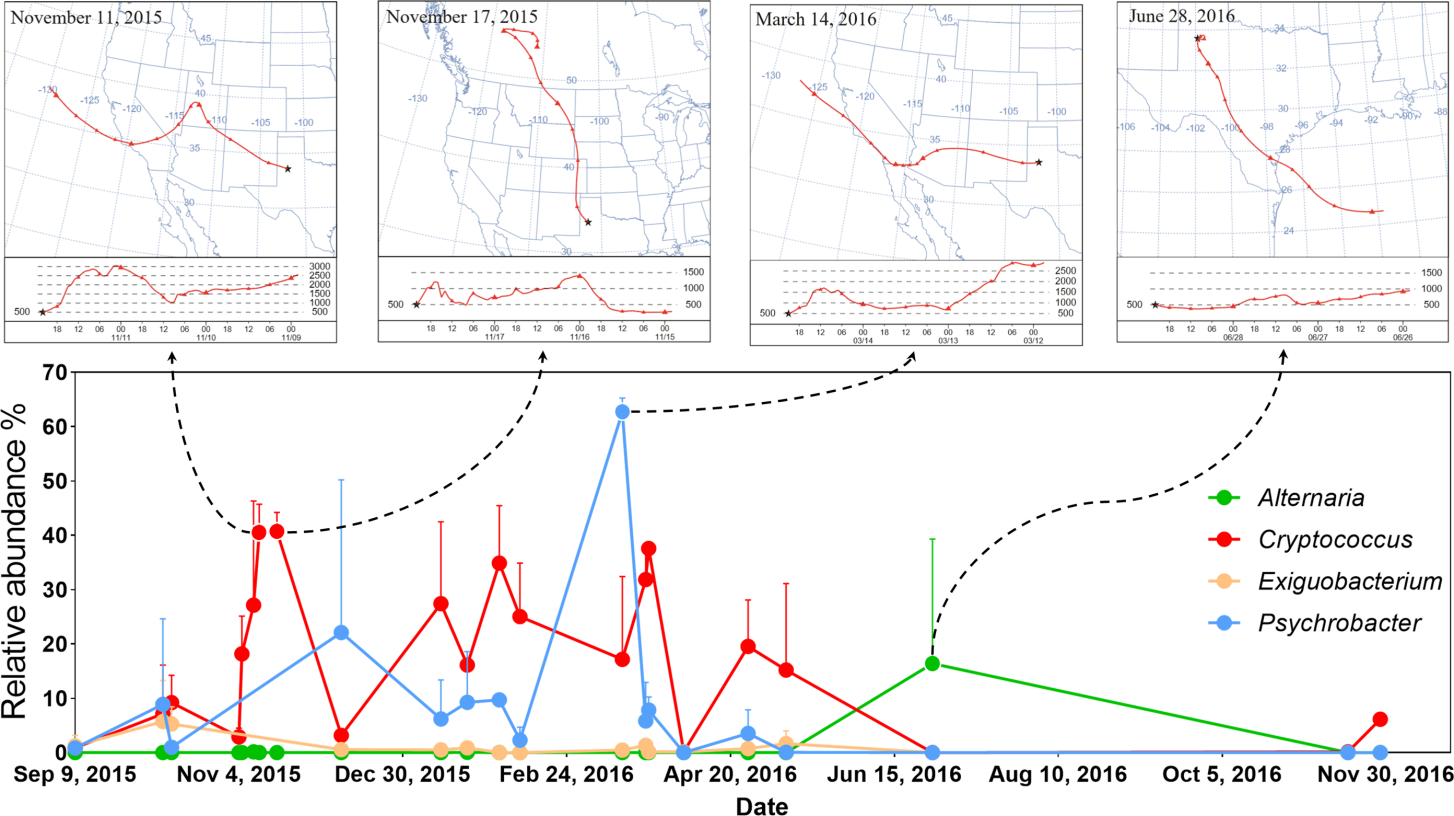
Correlations between microbiome composition temporal spike and origin of air parcels.

We unexpectedly observed psychrophiles belonging to the genera *Psychrobacter* and *Exiguobacterium* in a number of our samples. Examination of the back trajectories (Fig. 6) revealed that these genera were most abundant in air parcels that originated in the North Pacific or Northern Canada and when the elevation of the parcel was above 2000 m (Figure S2, Supporting Information). *Psychrobacter* was among the three most abundant bacterial genera observed over the entire collection period (Fig. 2B).

## DISCUSSION

This study emanated from an earlier qualitative observation of a large diversity of bacterial and fungal species in the atmosphere of the Texas Tech University campus during a very windy day in the spring of 2014. This area is among those with frequent dust storms in the United States due to the persistent winds (Orgill and Sehmel 1976; Deane and Gutmann 2003). As a region used for cotton, sorghum and grape growing, as well as livestock rearing, large areas of land may be left fallow for significant periods of time. Approximately 3.7 million acres of land is devoted to cotton cultivation annually in the Lubbock area high plains. These large tracts of land can contain dust particles that may be lifted up from the surface by winds. Particulates in the wind have been associated with respiratory ailments such as allergies and asthma (Hanson *et al*. 2016; Gat *et al*. 2017). In this study, we sampled air on the Texas Tech University campus over the 2015 and 2016 calendar years to assess the composition and relative abundance of bacteria and fungi based on calm and dust storm days.

Airborne particulates have increasingly been observed to spike during dust storms and act as carriers of bacteria and fungi, resulting in an increase in the microbial burden as well (Nourmoradi *et al*. 2015). Correlations between dust storms and the incidence of respiratory diseases have been long recognized (Hefflin *et al*. 1994; Kim, Kabir and Kabir 2015). A link between exposure to PM and hospital visits due to upper respiratory ailments has been observed by others (Tian *et al*. 2019; Ma *et al*. 2020), in some cases, a link was established between the long-term exposure and mortality rate in elderly (Wu *et al*. 2020). A recent study suggests that severe air pollution (enhanced PM_2.5_ levels as well as nitric oxide) may potentiate SARS-CoV-2 infections (Frontera *et al*. 2020). While the ubiquity of bacteria and fungi in the atmosphere have been assessed in different environments, the primary focus of previous studies has been on bacteria and not the fungi. Studies on atmospheric fungal microbiomes have largely focused on agricultural regions and practices or built environments (Dietzel *et al*. 2019). Abdel-Hafez *et al*. (1990) studied the fungal microbiome of wheat and sorghum harvester dusts and reported the common occurrence of *Alternaria, Aspergillus, Fusarium* and *Penicillium* species. Among these, many thermophilic taxa were also detected (Abdel-Hafez *et al*. 1990). In one study that assessed aerial transport of bacteria from cattle feed yards on PM in the area surrounding Lubbock, Texas, the authors identified antibiotic resistance genes present in these bacteria. Genes encoding resistance to tetracycline were more abundant in particulates downwind of the feed yards compared with collections made upwind (McEachran *et al*. 2015). Seasonal variations were correlated with changes in the airborne microbiome in agreement with atmospheric circulation (Cáliz *et al*. 2018).

Our study focused on airborne fungi and bacteria in an urban environment. We investigated the longitudinal abundance and diversity of fungal and bacterial genera and related these observations to the origin of the wind using back trajectories. Fungal genera and species present in our sampling with high relative abundance included *Aureobasidium pullulans, Cryptococcus consortionis, Alternaria, Cladosporium flabelliforme* and *Filobasidium*. These taxa belong to two phyla, Basidiomycota and Ascomycota. *A. pullulans* is a ubiquitous fungus that can cause several forms of infections in humans (Oliveira *et al*. 2013). Species of *Aureobasidium* have been observed in dust events in Africa (Griffin *et al*. 2003), sulfur-contaminated soils (Wainwright 1978), vineyards (Bozoudi and Tsaltas 2018) and were occasionally associated with *Cryptococcus* in vineyards (Comitini and Ciani 2008). Several *Cryptococcus* spp. were identified in this study. This genus contains species that are commonly associated with feces of birds (Kwon-Chung *et al*. 2014), particularly pigeons and canaries. The great abundance of pigeons on the Texas Tech University campus was very likely an influence on the *Cryptococcus* sampling. Species of *Cryptococcus* have been found to be sensitive to high temperatures but can tolerate alkaline soils, similar to those found in the Lubbock area. Species of *Cryptococcus* species, e.g. *C. neoformans* and *C. laurentii*, have been associated with human diseases (Byrnes *et al*. 2011; Molina-Leyva *et al*. 2013). Other spp., e.g. *C. infirmo-miniatus*, have also been used in the control of plant disease as part of integrated pest management (Spotts, Cervantes and Facteau 2002). *Cladosporium flabelliforme* is among the *Cladosporium* species that have been associated with clinical samples (Sandoval-Denis *et al*. 2015). The most interesting species of *Cryptococcus* we identified was *C. consortionis* first described in 1985, isolated from the Ross Desert of Antarctica (Vishniac 1985). Indeed *C. consortionis* was the second most abundant species of all the fungal samples identified in this study (Figs 1B and 6).

Mortierellomycota and Mucoromycota were the least represented fungi in our dataset. They were only identified in small number of days and at much lower abundance than other phyla. In the Mucorales order (belongs to Mucoromycota) several species are pathogenic and can cause skin and lung infections (Tomecki *et al*. 1989). Intriguingly, Mucorales-associated infections were found to be prevalent after natural disasters such as hurricane Katrina and the 2004 Indian Ocean tsunami (Kontoyiannis 2012). The reason behind the underrepresentation of these fungi in our dataset is that Lubbock area is not impacted by any hurricane air flow. Furthermore, the wind currents for most of the samples in our study originated in the higher latitudes (greater than 35°N latitude) and the northern Pacific Ocean.

*Coccidioides immitis*, the cause of the valley fever, is endemic in the southwestern parts of the United States, including Texas (Thompson *et al*. 2019). Interestingly, we did not detect *C. immitis* at any abundance in any day of our study. But we cannot definitively exclude the possibility of its presence as it could be a limitation of our culturing step.

The most predominant bacterial genera were *Bacillus, Pseudomonas, Psychrobacter, Massilia* and *Exiguobacterium*; of these, only members of the genus *Bacillus* form endospores. Endospores provide resistance to desiccation, temperature changes, chemical stress and have been known to survive for hundreds of years (Nicholson *et al*. 2000). Together with their ubiquity, it is not surprising that this genus is the most abundant, representing ∼50% of all genera. *Pseudomonas*, a bacterium with a large genome is ubiquitous and is well known for inhabiting a wide variety of habitats from soils and plants to hospital environments and is an opportunistic pathogen (Moradali, Ghods and Rehm 2017). Members of this genus are also known for their resistance to a broad variety of environmental chemicals and antibiotics and being excellent competitors by producing antimicrobial chemicals (Moradali, Ghods and Rehm 2017). *Pseudomonas* is also known to be an ice nucleator in clouds (Maki *et al*. 1974; Du *et al*. 2017). The abundance of both *Bacillus* and *Pseudomonas* species in our analyses is, therefore, not surprising. *Massilia* species have been isolated from different environmental samples (e.g. soil, air and water) and some of them have been associated with human infections (Peta, Raths and Bücking 2019). The occurrence of the two abundant genera, *Psychrobacter* and *Exiguobacterium*, was unexpected. Members of the *Psychrobacter* are known to be cold-loving and are often found in glaciers (Rodrigues *et al*. 2009). A few species of this genus are found associated with wound infections (Caspar *et al*. 2013). *Psychrobacter* was also in high abundance when wind elevations exceeded 2500 m or originated from the North Pacific Ocean (9 December 2015 and 14 March 2016). Species in the genus *Exiguobacterium* are known to have broad temperature tolerances (−12 to 55°C), and are also found in Himalayan soils, permafrost in Siberia and hot springs (White *et al*. 2018). The relative abundance of *Exiguobacterium* was highest on 12 October 2015 (9%), in contrast with an average of <1% across the rest of the days. When we observed the back trajectory maps (Figure S2, Supporting Information) we noticed that on this day, the air mass originated from the North Pacific Ocean.

The impact of dust storms on the atmospheric microbiome is of interest due to possible implications in human health and agriculture. The increase in the richness of the fungal atmospheric microbiome was notable as it resulted in the increase of several fungal genera. Among these was *Stemphylium vesicarium*, which is a plant pathogen and the cause of Welsh onion leaf blight (Aveling and Snyman 1993; Misawa and Yasuoka 2012). *Solicoccozyma aeria* (formerly *Cryptococcus aerius*) was isolated from soil samples and utilized in lipid production (Ghanavati, Nahvi and Karimi 2015). *Mortierella alpine* was another oleaginous fungus that we observed increased in dust storms days. *Naganishia albida* (formerly *Cryptococcus albidus*) has been associated with human infections (Lee *et al*. 2004). Other species of *Naganishia* have been collected in high elevation soils of the Atacama, Chile and Bolivia. These fungi also demonstrate high ultraviolet light resistance (Schmidt *et al*. 2017). *Rhodotorula graminis*, an endophytic yeast, is a carotenoid producer and plant growth-promoting fungus (Firrincieli *et al*. 2015; Lyman *et al*. 2019). *Vishniacozyma victoriae* (formerly *Cryptococcus victoriae*) was recently found to be prevalent in homes, especially with dogs present (Rush *et al*. 2020). Other species belonging to the genus *Vishniacozyma* have been identified from the Canadian High Arctic (Tsuji *et al*. 2019). The relative abundance of *C. consortionis* was also very high on dust storm days.

*Cladosporium, Alternaria, Aspergillus* and *Penicillium* species are notorious allergens (Kurup 2003). Interestingly, only *Cladosporium* and *Alternaria* were detected at high relative abundance (>5% on average) and in all collection times. In contrast, *Aspergillus* and *Penicillium* were observed in a few days with a relatively low abundance (<0.5% on average). This is because *Aspergillus* and *Penicillium* are more common indoors, while *Cladosporium* and *Alternaria* are ubiquitous both indoors and outdoors (Bozek and Pyrkosz 2017). The prevalence of these airborne allergens has an important implication in the health of asthmatic patients. For example, patients who are allergic and exposed to *Alternaria* have frequent exacerbations of their asthma that require hospitalization (Kołodziejczyk *et al*. 2016). Moreover, because dust storms are associated with increased asthma hospitalizations (Kanatani *et al*. 2010), we were interested to test the hypothesis that allergenic molds are increased in the air during dust storms and may contribute to this. Our results revealed that *Cladosporium* was the only mold (among the four allergens: *Cladosporium, Alternaria, Aspergillus* and *Penicillium*) that was increased during dust storms. This is an exciting result that requires further investigation, to find out whether the patients admitted to hospitals due to asthmatic complication (after a dust storm event) are allergic to *Cladosporium*. This knowledge can help susceptible patients and the healthcare system to avoid overburden after dust storms.

Among the bacterial taxa, *Stenotrophomonas* was increased in dust storm days and its species *S. maltophilia* is an emerging respiratory pathogen (Brooke 2012). *Paenibacillus* is one of the endospore-forming bacteria and its species is relevant to human health and agriculture (Grady *et al*. 2016). *Rheinheimera soli* findings are of interest in that it was significantly increased in dust storm days (i.e. it was only detected in dust storm days). *Rheinheimera soli* was first isolated from a soil sample in South Korea (Ryu *et al*. 2008). Other related *Rheinheimera* species were isolated from aquatic or marine habitats (Brettar, Christen and Höfle 2002; Romanenko *et al*. 2003). Linking the abundance of this fungus to back trajectory data showed that it had an abundance of 13% on 23 March 2016, when the wind originated from the North Pacific Ocean and reached an elevation of over 4000 m.

This research contributes to our broader understanding and efforts to appreciate the immense diversity of the Earth's microbiome (deciphering bacterial and fungal distribution), and efforts to harness and manage it (Alivisatos *et al*. 2015). Our study reveals that broad microbial diversity of fungi and bacteria in local environments is influenced by dust storm events, regional anthropogenic practices such as farming and the origins of air parcels. Interdisciplinary research linking microbiome, wind trajectory and environmental information with medical and public health data will enable researchers to establish a comprehensive epidemiological analysis of outbreaks to better understand their etiology and broad impacts on human, plant and wildlife health.

## ACKNOWLEDGEMENTS

We would like to thank the Departments of Biological Sciences and Geosciences for their support, and the Graduate School for support of MME.

## Supplementary Material

xtaa009_Supplemental_File
