# Supplementary material for: Investigating the culturable atmospheric fungal and bacterial microbiome in West Texas: implication of dust storms and origins of the air parcels
Source: FEMS Microbes. 2020 Dec 15;1(1):xtaa009. doi: 10.1093/femsmc/xtaa009 (PMC10117434; doi:10.1093/femsmc/xtaa009)
Supplement: xtaa009_Supplemental_File [file xtaa009_supplemental_file.docx]

# **Supplementary Tables**

**Supplementary Table S1. Meteorological information on sample collection days.**

| **Date** | **Dust Storm observed** | **Wind speed (km/h)** | **Wind Gust (km/h)** | **Visibility (km)** |
| --- | --- | --- | --- | --- |
| 9/9/2015 | No BLDU^1^ | 17.7 | NA^2^ | 16.1 |
| 10/9/2015 | No BLDU | 17.7 | 32.2 | 12.9 |
| 10/12/2015 | No BLDU (Calm) | 27.4 | 45.1 | 16.1 |
| 11/4/2015 | No BLDU | 32.2 | 46.7 | 16.1 |
| 11/5/2015 | No BLDU | 45.1 | 61.1 | 12.9 |
| 11/9/2015 | No BLDU (Calm) | 29.0 | 38.6 | 16.1 |
| 11/11/2015 | No BLDU | 38.6 | 49.9 | 16.1 |
| 11/17/2015 | No BLDU | 54.7 | 74 | 16.1 |
| 12/9/2015 | No BLDU (Calm) | 22.5 | NA | 16.1 |
| 1/12/2016 | No BLDU (Calm) | 17.7 | NA | 16.1 |
| 1/21/2016 | BLDU | 46.7 | 64.4 | 16.1 |
| 2/1/2016 | BLDU | 53.1 | 74 | 4.8 |
| 2/8/2016 | No BLDU (Calm) | 22.5 | NA | 16.1 |
| 3/14/2016 | BLDU | 41.8 | 72.4 | 16.1 |
| 3/22/2016 | BLDU | 49.9 | 66.0 | 12.9 |
| 3/23/2016 | BLDU | 54.7 | 75.6 | 6.4 |
| 4/4/2016 | No BLDU (Calm) | 22.5 | NA | 16.1 |
| 4/26/2016 | BLDU | 33. 8 | 49.9 | 11.3 |
| 5/9/2016 | No BLDU | 46.7 | 69.3 | 11.3 |
| 6/28/2016 | No BLDU (Calm) | 24.1 | NA | 16.1 |
| 11/17/2016 | No BLDU | 33. 8 | 49.9 | 16.1 |
| 11/28/2016 | No BLDU | 45.1 | 62.8 | 16.1 |

^1^ BLDU—Dust storm event

^2^ NA—Not available

**Supplementary Figures**


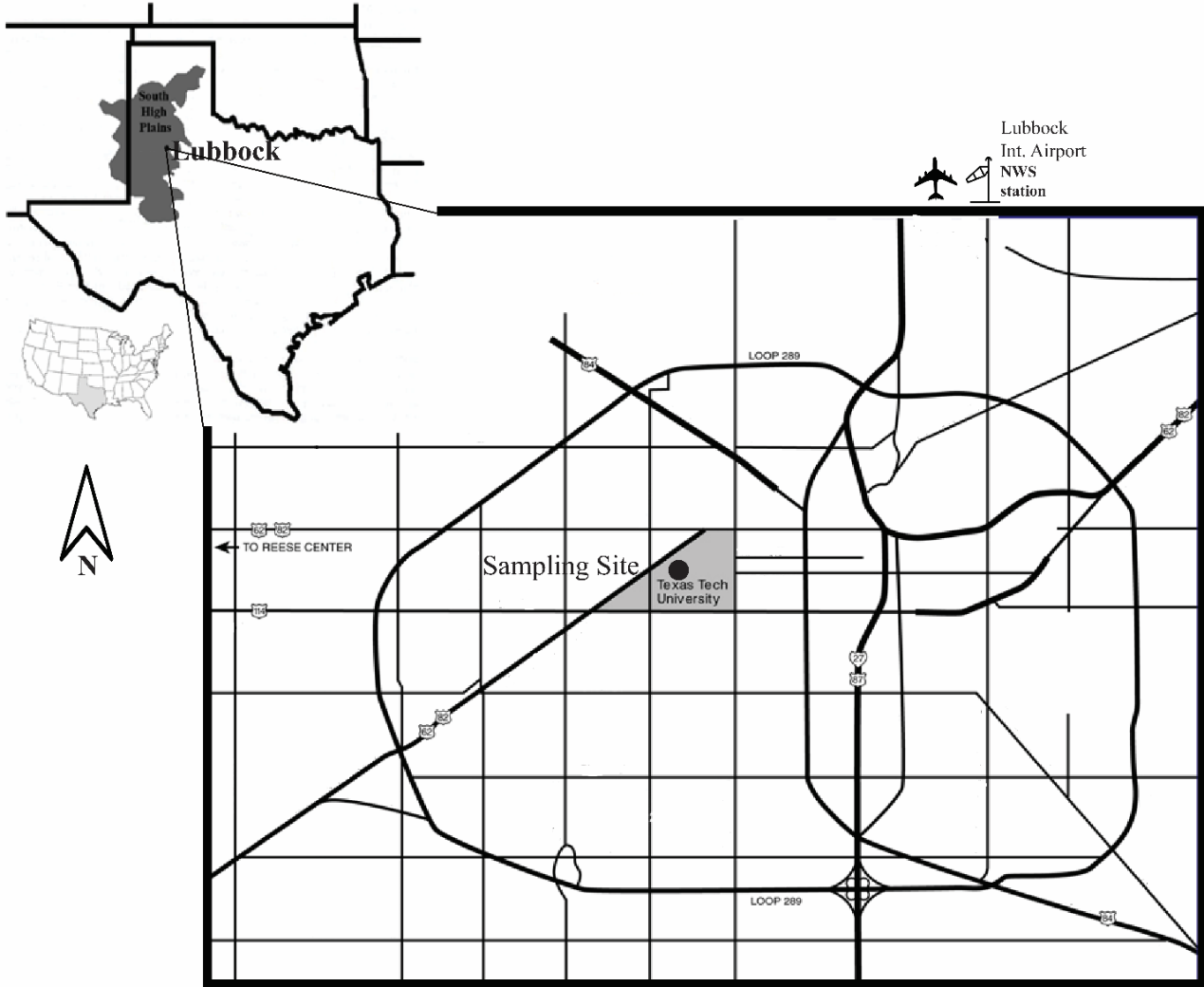


**Supplementary Figure S1.** Location of Lubbock, the NWS meteorological and the collection site on Texas Tech University.


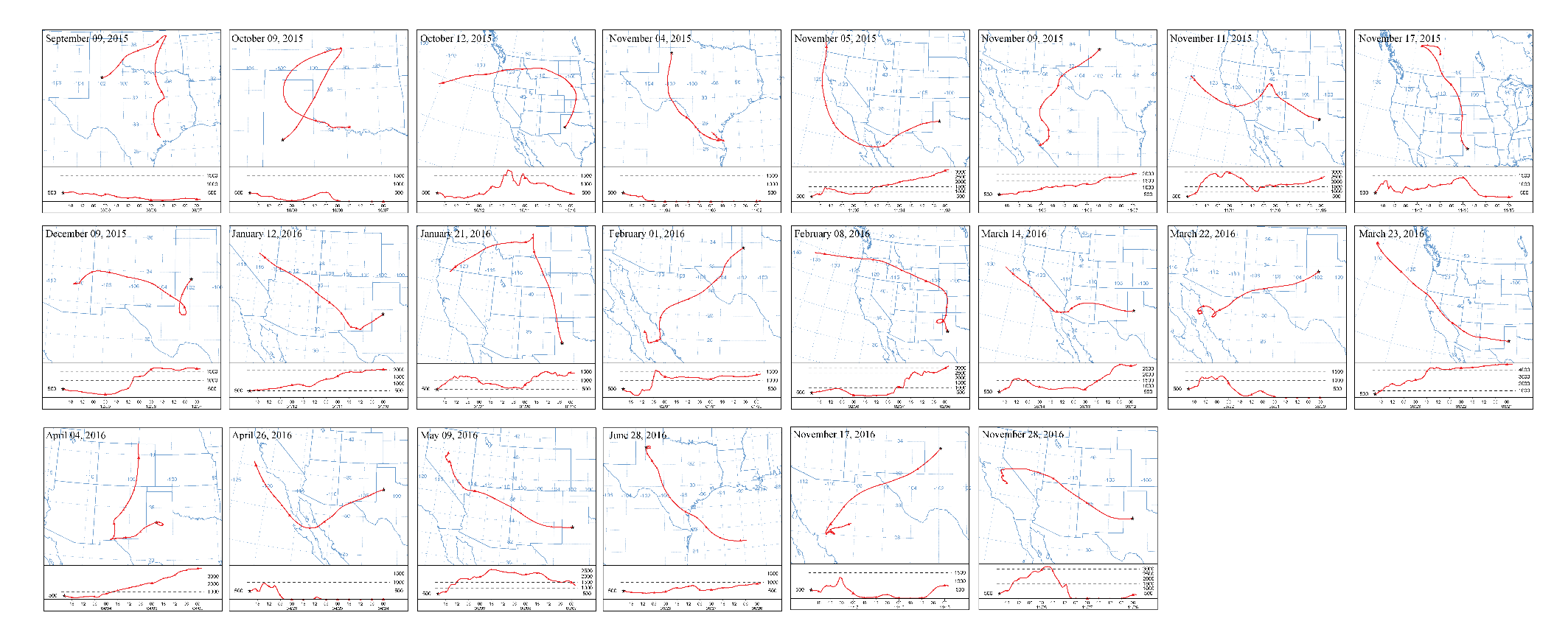


**Supplementary Figure S2.** Back trajectory analyses depicting the origin of the air mass on sampling days over 72-hour periods from the coordinates of the sampling site in Lubbock, Texas.
